# Dogs in the Workplace: A Review of the Benefits and Potential Challenges

**DOI:** 10.3390/ijerph14050498

**Published:** 2017-05-08

**Authors:** Anne M. Foreman, Margaret K. Glenn, B. Jean Meade, Oliver Wirth

**Affiliations:** 1 National Institute for Occupational Safety and Health, Centers for Disease Control and Prevention, Morgantown, WV 26505, USA; oaw5@cdc.gov; 2 College of Education and Human Services, West Virginia University, Morgantown, WV 26505, USA; Margaret.glenn@mail.wvu.edu; 3 Davis College of Agriculture, Natural Resources and Design, West Virginia University, Morgantown, WV 26505, USA; jmeade@cheatlakevets.com

**Keywords:** animal-assisted activities, animal-assisted interventions, pet effect, mental health

## Abstract

Pet dogs, therapy dogs, and service dogs can be seen in workplaces with increasing frequency. Although dogs may provide many benefits to employees and employers, their presence may introduce additional hazards and concerns to the work environment. Therefore, decisions to accept dogs in the workplace may include many considerations including the health, safety, and well-being of employees, legal and cultural sensitivities, and animal welfare. The present paper serves to introduce the issue of dogs in the workplace and outline the potential benefits and challenges to their presence. The legal accommodations afforded to certain types of dogs in workplace settings are discussed, and the research findings pertaining to the potential benefits of dogs on human health and well-being are summarized. The paper concludes with considerations for human resource management personnel in the areas of diversity, employee relations, ethics and corporate responsibility, organizational and employee development, safety and security, and legal considerations, as well as suggested topics for future research.

A survey by the American Veterinary Medical Association revealed that there were approximately 70 million pet dogs and 74 million pet cats in the United States in 2011 [1]. Sixty-three percent of those surveyed considered their dog or cat to be a part of their family. Dogs are no longer confined to the home environment; they are also appearing in increasing numbers in the workplace for assistance with a disability, emotional support, or even companionship [2]. For example, prominent companies, such as Google and Amazon, allow employees to bring their pet dogs to work [3], and the Friday following Father’s Day each year has been designated “Take Your Dog To Work Day” since 1999 [4]. The 2015 Society for Human Resource Management’s Employee Benefits survey found that 8% of respondents reported that their workplaces permitted pets, an increase from 5% in 2013 [5]. In addition to pet dogs, service dogs may also be present in work environments to assist individuals with a disability. Despite the increasing prevalence of dogs in the workplace, there is a dearth of scientific evidence regarding the impact of dogs on performance, employee relations, workplace culture, and worker health and safety. The present paper addresses the potential benefits, concerns, and challenges of accommodating dogs in the workplace and highlights several factors and requisite conditions that can be considered by employers, employees, and human resource personnel when deciding to accept a dog in the workplace. Gaps in current knowledge, directions for future research studies, and considerations for human resource personnel are also addressed.

## 1. Types of Dogs in the Workplace

Dogs may be present in contemporary work settings for various reasons, but it is most common perhaps to see a service dog accompany an employee with a disability. According to the Americans with Disabilities Act (ADA) Titles II and III, service dogs are individually trained to perform work or tasks to assist an individual with a disability [6]. The nature of the disability may be physical, as with a mobility, visual, or hearing impairments, or it may be psychological, as with post-traumatic stress disorder (PTSD) or panic disorder. In cases involving a physical disability, a service dog may be trained to help an individual use a wheelchair, retrieve items, open doors, navigate corridors and streets, alert for alarms, and so on. With psychological disabilities, such as panic disorder, a service dog may be trained to perform tasks that have therapeutic benefits, such as lying across an individual’s lap during a panic attack to reduce the duration of the attack and associated symptoms of anxiety. According to federal law, service dogs constitute a reasonable accommodation to an individual with a disability in employment settings under Title I of the ADA [6]. An emotional support animal, a pet that is not specially trained but provides emotional support to an individual with a disability, may also constitute a reasonable accommodation in the workplace under Title I.

A visitation dog is another type of dog frequently seen in work settings. Visitation animals, in general, refer to animals that accompany their owners on visits to healthcare and educational settings to socialize with patients or students [7]. Visitation animals are usually dogs, but they can also be other animals including but not limited to cats [8], birds [9], and horses [10]. It is common to see visitation animals in healthcare settings, such as pediatric hospitals [11,12,13,14], psychiatric hospitals [15,16,17,18,19,20], palliative care centers [21,22,23,24], and nursing homes [25,26]. In educational settings, visitation animals are often present in elementary schools [27,28,29,30] colleges [31,32], and libraries [33,34,35]. In most cases, visitation animals are present in a setting for a few hours a day or week, but they may also be a constant presence. Resident dogs may live in a facility or come to work every day with an employee [36,37]. Resident dogs are common in nursing homes, child advocacy centers, and courtrooms [38].

Therapy dogs can also be found in workplaces. A therapy dog assists a professional service provider (often in a healthcare setting) in the treatment or rehabilitation of a patient or client [7]. These dogs can be found accompanying occupational therapists [39,40], psychotherapists [41], speech-language pathologists, physical therapists [42] and other professionals in the performance of their job duties. In an occupational therapy setting, for example, a therapy dog can help improve a patient’s gross motor skills through encouraging exercises such as grooming the dog, throwing a ball, or walking with assistance from the dog [43]. In a psychotherapy setting, the dog may help the therapist develop rapport with a client [44] or facilitate child therapy [45].

In other cases, employers are establishing pet-friendly workplaces that permit well-behaved dogs regardless of an employee’s disability status. For example, Google, Amazon, and Etsy have well-publicized policies permitting their employees to bring their dogs to work. In some cases, on-site accommodations are provided for care and welfare of the dogs, including dog parks [46]. At the Seattle headquarters of Amazon, for example, there are dog-sized water fountains next to the normal fountains, containers of dog treats at the reception desks, and waste-bag dispensers outside along the paths between buildings [47].

Although they may be present in some workplaces, this review does not include some categories of assistance animals including public or military service animals (e.g., police dogs, military working dogs, search-and-rescue dogs, etc.) or sporting, recreational, agricultural activity animals (e.g., herding dogs, livestock guard dogs, etc.) [7]. These types of assistance animals have limited interactions with the public or spend limited time within places of business. For example, police dogs spend the majority of their time with their primary handlers and not in office settings [48]. Additionally, policies and procedures for the care and training for many types of public or military service animals have already been developed by city and state police departments [49,50].

## 2. Accommodating Dogs in the Workplace

When considering the prospect of dogs in the workplace, it is important to know in which situations dogs are discretionary and in which situations dogs are a legal accommodation. Employers are obligated to allow dogs only in select circumstances [51]. An employee generally does not have a legal argument for bringing a dog to work other than as an effective accommodation solution for a functional limitation associated with a disability. According to Title I of the ADA [6], employers must make “reasonable accommodations,” in the workplace for individuals with disabilities. A reasonable accommodation is defined as a modification or an adjustment to a job or the work environment that allows an employee with a disability to perform essential job functions [52]. Service dogs (and even emotional support animals, because there is no specific definition of service animal under Title I) can be considered a reasonable accommodation, however, the term reasonable is important in this discussion. A service dog is not permitted into a workplace unconditionally. Employers may have the right to deny a service dog access to the workplace if the dog constitutes an “undue hardship,” defined as an act that requires significant difficulty or expense or fundamentally alters the nature of the business [52]. For example, if an employee working in the clean room of a semiconductor fabrication plant requests that she be allowed to bring her service dog to work with her, the employer may consider this accommodation an undue hardship because the presence of the dog would fundamentally alter or compromise the dust-free manufacturing environment.

The broader issue of access and the law is an important consideration for employers because the needs of the individual with the service dog are often weighed against potential adverse effects of the presence of the dog. For example, consider a situation in which an employee with a service dog brings the dog to an office setting as a reasonable accommodation for a disability, and a co-worker in an adjacent cubicle has a severe allergy to dogs. Because the dog is a service dog, and unless the accommodation constitutes an undue hardship to the business, the employer will consider reasonable accommodations which may include, for example, modifying the work environment (e.g., moving one employee to an alternate work site) or rearranging work schedules (e.g., permitting the employees to work alternate shifts or telework). It is important to note that the ADA only applies to a service dog or other animal that assists a person with a disability in eliminating or mitigating barriers to completing essential work tasks. If the dog is a visitation therapy dog or a pet, employers are not obligated to make any accommodations, and thus the dog could be banned from the workplace. Awareness and understanding of the legal obligations of employers under the ADA and relevant state laws are critical to ensuring that both the employee with a disability and co-workers are treated fairly when accommodations are considered and implemented [53]. For example, according the Revised Code Title 49 Section 60.180 of Washington State, it is unfair to refuse to hire someone because they have a service dog or to fire someone because they obtain one. Other states specify provisions for the use of leave to acquire or train a service dog (e.g., Texas Government Code Title 6 Section 661.910).

## 3. Research on the Effects of Dogs on Mental Health and Well-Being in Humans

An employer’s decision to accept a dog in the workplace ought to take into account the potential impacts to the health, safety, and well-being of co-workers, regardless of whether the decision accommodates an employee with a disability or fulfills the employer’s desire to create a pet-friendly workspace. When an employee brings a dog to work, whether as a service dog or pet, the effects on the workplace extend beyond the employee. Indeed, the entire business milieu, including other co-workers, managers, maintenance and custodial staff, and customers or clients, may be affected by the presence of the dog. Some effects may be positive. For example, empirical evidence supports the notion that dogs may provide social support [54], improve performance, and increase social interactions. However, there may be other aspects to be considered related to health, safety, interpersonal, and cultural issues. Although there is a dearth of definitive research studies on this topic, researchers have studied the impact of dogs in the areas of social support and stress reduction, performance, health and safety, and social interactions. The following sections highlight major findings that may be taken into account in decisions to accommodate dogs in the workplace.

### 3.1. Social Support and Stress Reduction

One potential benefit of dogs in the workplace is that they provide an additional source of social support for the employee. The term “social support” is often used to describe the mechanisms by which relationships with other people buffer individuals from stress [55]. For example, increased social support was associated with lower levels of depression and better job performance in a survey of 240 hospital workers [56], whereas low social support at work has been associated with diagnoses of depression and anxiety [57]. In a qualitative study, social support was identified as a key factor in whether people with serious mental illness return to work or remain employed [58].

Researchers investigating the role of pets in buffering stress reported that pets may serve as a source of social support, perhaps more effectively than a spouse or close friends. For example, Allen et al. [54] compared the effects of the presence of a spouse or a family pet on individuals’ cardiovascular responses to a stressful task. Blood pressure and heart rate were recorded during a cold-pressor task (submerging a hand in ice water) and a mental-arithmetic task under one of three conditions: alone (control condition), in the presence of a spouse, or in the presence of a pet. During the pre-task baseline periods and the tasks, heart rate and blood pressure were significantly lower when a pet was present than when a spouse was present. Furthermore, performance of the mental-arithmetic task had the fewest errors in the condition with a pet present. Similar studies have found that the presence of a dog reduced skin conductance responses [59] and salivary cortisol [60] to a significantly greater degree than that of a human friend.

The nonevaluative (i.e., nonjudgmental) role of service dogs or pets may be an important contributing factor to their therapeutic effects. Although not directly studied with dogs, similar physiological effects have been obtained in studies with a friend present and when the experimental design limited the possible evaluative role by the friend. For example, Kamarck et al. [61] administered a mental arithmetic task and a concept formation task to 39 women while recording their heart rate and blood pressure. The women performed these tasks either alone or in the presence of a friend. To reduce the real or perceived evaluative role of the friend, the friend wore headphones that played white noise and filled out questionnaires during the tasks. The women in the friend group had significantly smaller increases in heart rate during both tasks than those who completed the tasks alone. For instance, during the mental arithmetic task, heart rate increased an average of 8 beats per minute for the friend group versus 18 beats per minute for the alone group. Although this experiment did not directly compare nonevaluative and evaluative support by the friend, the findings support the notion that the nonevaluative role of service dogs or pets may be an important contributing factor to their therapeutic effects.

Other studies comparing only the presence versus the absence of a dog have observed lower stress responses when the dog is present than when it is absent. For example, Friedmann et al. [62] used a quiet-talk-quiet (QTQ) protocol during which older adults with hypertension sat quietly for two minutes, talked to the experimenter for two minutes, and then sat quietly again for two minutes. The QTQ protocol was conducted in the presence and absence of a friendly, unfamiliar dog. On average, systolic and diastolic blood pressure was 7 and 2 mmHg lower, respectively, when participants spoke with the dog present than when the dog was absent.

Two studies have examined the effects of dogs in the workplace on stress and well-being. In one study of the effects of dogs in the workplace on employees’ self-reported stress, employees who did and did not bring dogs to work completed a perceived stress survey several times throughout the work day [63]. Employees who did not bring dogs to work had significantly higher perceived stress than employees who did. To assess differences in stress, employees who brought their dogs to work were instructed to leave them at home two days a week during the one-week study period. On days when employees in the dog group did not bring their dogs to work, their stress levels increased throughout the day, matching the pattern of employees who never brought dogs to work. In another study in which participants who did and did not bring their pets to work were asked about the psychological and organizational effects of dogs at work, the perceived benefits of dogs at work were greater for those who brought their dogs to work compared with those who did not bring their dogs and those who did not own pets [64]. Besides the apparent benefits of dog–human companionship at work, these results suggest that the perceived benefits of dogs in the workplace may also depend on an existing relationship with the dog present in the workplace. Indeed, the topic of dog-human relationships or bonding is receiving more research attention [65] and this work may have important implications for understanding the benefits of dogs to employees’ stress and well-being.

The aforementioned studies suggest that the presence of a dog is associated with reduction in physiological indicators of stress (even more effectively than friends and family in some cases) and improved performance, but other studies have not found such effects. In one study, 32 college students were asked to complete mental arithmetic problems and the Thematic Apperception Test (in which the subject interprets ambiguous pictures) while heart rate and blood pressure were measured [66]. Half of the students completed the tasks in the presence of their pet dogs, and the other half completed them alone. There were no statistically significant differences between the two groups for heart rate or blood pressure during the tasks. Other similar studies have failed to find significant differences in physiological measures of stress in the presence and absence of animals [66,67,68,69,70,71,72]. These inconsistencies in findings may be due to small sample sizes, the use of insufficiently stressful stimuli, or lack of control for other potentially influential factors. Additionally, in many studies, the physical movements involved in petting and talking to the animals may have increased the blood pressure and heart rates of the participants [69,71,72], obscuring any potential stress-relieving effects. It may also be the case, however, that dogs do not provide acute decreases in stress and improvements in performance.

### 3.2. Task Performance

Besides evidence of stress reduction in the presence of dogs, some studies also reported performance-related changes. A performance-enhancement effect of pets was found in a study of pets and cardiovascular reactivity. Allen et al. [73] randomly assigned forty-eight individuals with hypertension to a pet group or a non-pet group. Individuals in both groups began taking hypertension medication, but only those assigned to the pet group were instructed to obtain a pet. Both groups performed stress-inducing tasks (arithmetic and speech) before starting the medication and obtaining the pet and after six months. For the pet group, the tasks were administered with the pet present. Subjects who had acquired pets showed significantly greater improvements in their performance of the arithmetic and speech tasks compared with those who had not. There were also significant physiological differences between the groups at the six-month follow-up; those with pets had statistically significantly lower mean heart rates (79 vs. 88 beats per minute (arithmetic) and 79 vs. 93 beats per minute (speech)) and lower mean systolic (130 vs. 139 mmHg (arithmetic) and 126 vs. 139 mmHg (speech)) and diastolic blood pressure (90 vs. 95 mmHg (arithmetic) and 89 vs. 99 mmHg (speech)). The researchers frame these performance-enhancing and stress-decreasing responses in the presence of pets as a form of social support.

### 3.3. Social Interactions

Another potential benefit of dogs in the workplace is that they may have a positive effect on the social interactions among employees. Anecdotal reports suggest that pets enhance the social atmosphere at work [74], and research conducted outside of the workplace indicates that dogs can increase the frequency of conversations among people [75]. Other studies have been conducted to assess the role of dogs in changing the interactions between strangers and among groups of people who are familiar with one another.

Researchers have taken an experimental approach in assessing the role of dogs in changing social interactions with acquaintances and strangers. Several studies have shown that when an individual is accompanied by a dog, the frequency of social encounters with strangers increases. One such study compared the approaches of strangers when a female confederate (an actor who is part of the experiment and knows the aims of the study) was alone and when she was accompanied by different types of dogs (e.g., adult Rottweiler, adult Labrador retriever, or juvenile Labrador retriever), a teddy bear, or a potted plant [75]. Over 30% of strangers talked to the female confederate when she was accompanied by an adult or juvenile Labrador retriever compared to less than 5% of strangers when she was alone or accompanied by the teddy bear, potted plant, or Rottweiler. Another study had the confederate take on a more active role, either soliciting strangers for money, dropping coins on the ground, or asking young women for their phone numbers [76]. In all three scenarios, strangers were more receptive to the confederate when the dog was present than when it was absent. Additionally, studies conducted with individuals who use wheelchairs, a subset of the population who often benefit from service dogs, have found that strangers are more likely to engage in conversations with them when accompanied by a dog [77].

Because workers are more likely to encounter familiar co-workers and acquaintances than strangers, it is unclear whether the research on social interactions among strangers is relevant. To date, there have been no studies conducted on the presence of dogs in the workplace and its effects on the frequency of social interactions among employees; however, results from the animal-assisted activities literature may inform this unexplored research area. In animal-assisted activities, visitation therapy dogs are taken to nursing homes, hospitals, schools, and other settings to visit the residents, patients, or students [78]. Like co-workers in a workplace, patients in nursing homes and hospitals encounter one another on a daily basis.

In a typical research study examining the effects of an animal visitation program on social behavior (i.e., initiating conversation, smiling, laughing, etc.), interactions during animal-assisted activity sessions are compared with control sessions in which the dog is absent. In one nursing-home study, during 30-min sessions observers recorded the frequency of a variety of behaviors, including non-attentive behavior (e.g., sleeping or reading), attentive and non-attentive listening, and social behaviors towards other residents or towards the dog [79]. Verbal and non-verbal interactions towards other residents occurred twice as often when the dog was present than when it was absent. Similar studies also found increases in social interactions with the implementation of animal visitation programs [80,81,82].

One of the few studies that examined the perceptions of the impact of pets in the workplace supports the hypothesis that dogs can increase social interactions and improve mood. College students were presented with a picture of an office with a dog, cat, or no animal superimposed into the picture [83]. They were asked to imagine that they were employees in this office and then answer several survey questions about employee satisfaction and mood. Students who viewed the image that included a dog or cat perceived that their mood would be better and that there would be a greater number of social interactions than those students who viewed the picture without an animal. In a similar study by the authors, college students rated a picture of a professor’s office with a dog, cat, or no animal superimposed onto it [2]. When there was a dog in the office, students perceived the professor to be friendlier than when a cat or no animal was present.

One potential disadvantage of increased social interactions among employees is the possible distraction from an individual’s work tasks. Based on the results of the studies described above, it is plausible to conclude that a dog in a workplace might invite unsolicited social attention from other employees, which in turn serves as a distraction from work tasks. It is also possible that the initial novelty of having a dog in the workplace may lead to a temporary decrease in productivity, but, as employees habituate or become accustomed to the dog’s presence, the level of distraction may subside. We are not aware of any research studies on the impact of dogs on workplace productivity.

### 3.4. Limitations of Research Evidence

Although there has been a great number of research studies conducted in the area of human–animal interactions, there are important caveats to the conclusions that can be drawn from the results. For example, studies on the effects of dogs on social interactions and stress primarily consisted of laboratory studies. The generalizability of laboratory studies can be limited due to the contrived nature and high degree of control in laboratory settings; therefore, the results of these studies should be interpreted with caution. Most of the studies also consisted of single, one-hour or shorter sessions, and thus long-term effects are unknown. It is possible that the short-term reductions in stress are transient or disappear altogether after frequent or regular exposure to a dog in the environment.

Methodological limitations are characteristic of other studies, especially those conducted on animal-assisted interventions and activities in healthcare settings (e.g., nursing homes). The common methodological shortcomings in these studies have been discussed elsewhere [84,85,86]. For example, many studies fail to include control groups, randomize group assignment, standardize the therapy sessions, and collect measures of inter-observer agreement [84]. Until methodologically stronger studies are conducted, the conclusions drawn from studies that have been conducted should be tentative at best.

## 4. Health, Safety, and Well-Being Issues

Whereas dogs may provide many benefits to people in the workplace, their presence may also pose several hazards and risks. Some of these hazards and risks are related to health concerns that would accompany non-human animals into any setting, including allergies and animal-borne diseases (i.e., zoonoses). Other hazards and risks are related to safety concerns, like slip, trip, and fall hazards and dog bites. Other issues pertain to employees’ psychosocial and cultural well-being.

### 4.1. Allergies

An estimated 15–30% of people with allergies have allergic reactions to dogs and cats [87]. Even when dogs are not present, studies have shown that canis familiaris 1 (a major dog allergen) is pervasive in public places [88]. Allergic reactions to dogs vary in intensity, and common symptoms include swelling and itching of the membranes that line the eyes and nose, breathing problems, and a rash on the face, neck, or chest [87]. Therefore, the threat of allergic reactions to the presence of dog in the workplace is a real concern that faces employers, health and safety personnel, and human resource staff.

Various methods exist to effectively reduce allergens in workplaces that allow dogs. The methods have been implemented and used effectively in other workplaces that involve human–animal contact, such as research laboratory workspaces [89]. Common methods include surveying employees to assess existing allergies, limiting exposures to the animals, or providing sensitive employees with personal protective equipment. Given that the major dog allergens are found in dander [90], workplace exposure to allergens could be reduced by asking the dog owners to bathe their dogs regularly or before bringing them in, as twice-a-week bathing has been shown to reduce allergens [91,92]. If the dog present in the workplace is a service animal, this may be an unreasonable request as it would likely place an undue burden on the handler with the disability. Air cleaners with high-efficiency particulate air (HEPA) filters have been shown to reduce the amount of dog dander in the air [93]. Unsurprisingly, airborne dog dander levels are much lower when the dog is not in the room [94] therefore, designating “dog-free” areas is an additional method that could be used to reduce dog dander in workplace settings.

### 4.2. Zoonoses

The potential transmission of zoonoses is another health concern in the workplace. A zoonotic disease is an infectious disease that can be transmitted between non-human animals and humans. Types of zoonoses common in dogs include internal and external parasites, viruses, bacteria, and fungi [95]. Zoonoses can be spread through direct contact (e.g., via feces, urine, skin, respiratory secretions) or indirect contact (e.g., via water or food that has been contaminated by the animal) [95]. As with many infectious diseases, children and immunocompromised individuals are at greater risk for contracting zoonoses [96].

Controlling potential zoonoses associated with dogs in public settings is an ongoing concern. Companion animals have been present in nursing homes and hospitals as part of animal-assisted therapeutic activities for decades, and most institutions have developed and implemented policies to help reduce the risks of zoonoses in these settings. These policies typically include instructions for frequent hand washing, regular deworming and vaccination of the animals, and prompt removal of animal waste [97]. For example, the University of Texas Medical Branch has a detailed policy for animal-assisted therapy visits, including a specific protocol that includes checking the visiting animal for the presence of bandages, signs of gastrointestinal disease, and respiratory disease [98].

### 4.3. Slip, Trip and Fall Hazard

Potential hazards of pets in the workplace also include environmental safety hazards. For example, because dogs are low to the ground and often attached to leashes, dogs constitute both direct and indirect fall hazards [99]. Dogs can cause falls directly when they pull an individual on a leash or when someone trips over a dog lying on the floor. Indirect causes of falls occur when an individual trips over a dog’s chew toy or slips in a puddle of water from a dog bowl spill. In 2006, falls associated with dogs (not restricted to workplace exposure) and cats resulted in an estimated 86,000 injuries in the United States [100]. According to studies that collected data on emergency room visits, the highest injury rates occurred among people over 75 years of age, and the most common injury was fracture [99,100].

The risks of both indirect and direct causes of falls can be reduced with education and practice considerations. The trip-and-fall risk associated with dogs could be communicated to all employees through training to make employees cognizant of the potential hazard [99], leash lengths could be limited to 4–6 feet to prevent falls associated with long or flexible, extendable leashes stretched across hallways or rooms, and dog toys and bowls could be placed in designated spaces, away from frequently trafficked areas. Individuals bringing their therapy dogs or pet dogs to work can be notified of such protocols for reducing fall hazards and penalized for protocol violations. Note that service dogs are typically trained to lie under desks and tables, away from doorways and hallways [101], minimizing the risks.

### 4.4. Dog Bites

One of the most serious risks of permitting dogs in the workplace—and one of the most serious concerns for the employer—is bites to employees or customers. According to some estimates, dogs are most frequently responsible for animal bites to humans (80–90% of mammalian bites), followed by cats (5–15%) [102,103]. Dog bites can range in severity from light bruising to serious lacerations, tears, and crushing injuries. Most dog bites are not severe enough to require a hospital visit—only approximately 17–18% of dog bites receive medical attention [104].

Although any dog is capable of biting, the dogs most often present in workplaces (e.g., those selected for visitation therapy dog or service work) should have been extensively evaluated and tested for calm, non-aggressive temperaments. In 1996, The Delta Society (now Pet Partners), a non-profit organization that facilitates human–animal interactions for the benefit of human health, developed standards of practice for the selection of visitation animals [105]. Other organizations that evaluate and register visitation dogs, such as Therapy Dogs International, also have strict testing protocols for evaluating potential therapy dogs [106]. These tests or standards are similar to the Canine Good Citizen Test created by the American Kennel Club. To pass the Canine Good Citizen Test, the dog must be able perform basic obedience tasks, such as walking calmly on a leash, greeting a friendly stranger, and coming when called [107]. If a dog shows any signs of aggression during testing (e.g., growling, snarling, barking) the test ends and the dog does not pass.

An important consideration for employers is their legal responsibilities regarding dogs in the workplace in the event that a dog injures an employee. Dog bite laws vary from state to state, and the definition of owner or keeper of the dog is particularly important for employers. For example, in the Oregon statutes, an owner is defined as “*any person having a right of property in an animal or who harbors an animal or who has it in the care of the person, or acts as its custodian, or who knowingly permits an animal to remain or about any premises occupied by the person.*” [108]. Theoretically, an employer could be personally liable as an owner or keeper of an employee’s dog in the workplace. In a court case in Connecticut, the employer was not found to be a keeper. In LaVoy v. Rosenthal, in which the business owner’s dog bit an employee (LaVoy), the judge found that because the dog did not aid in the business operation and no other employee had dominion or control over the dog as a part of their work duties, the corporation was not considered a keeper of the dog [109]. In a similar case, the court found that the employer was not a keeper under the state’s strict liability dog-bite statute, but the employee could still bring a claim of negligence against the employer for the injury that occurred at the work site [110]. Given the large amount of variability in state, county, and community statutes and regulations, it is important to be knowledgeable about relevant laws in an employer’s location and to ensure that appropriate insurance is carried by all parties.

### 4.5. Fear and Phobias

Some employees may be uncomfortable with a dog in the workplace because they are afraid of them. A Gallup poll conducted in 2001 found that 11% of Americans surveyed were afraid of dogs [111]. The intensity of the fear or anxiety responses can be mild or severe, as in a phobia. A dog phobia is a type of animal-specific phobia, defined as a marked fear or anxiety about the animal at levels that are out of proportion to the actual danger, active avoidance of the animal, and clinically significant distress or impairment in social, occupation, or other areas of functioning. For someone with a dog phobia, responses including fear, anxiety, and even panic may be triggered by the mere presence of a dog or even images or thoughts about dogs [112]. Animal-specific phobias have a prevalence rate in the general population that ranges from 3.3% to 7%, and the rate is similar across people of all ages [113].

### 4.6. Cultural Sensitivities

In addition to dog fears and phobias, it is important for employers to consider the perceptions and attitudes of employees regarding dogs in the workplace. Perceptions of dogs can differ appreciably across different societies and cultures. For example, using dogs as a food source is a historical and cultural practice in some countries such as South Korea and Vietnam [114], but it remains taboo in the United States and other western cultures where dogs are kept only as pets. In some cultures, dogs are considered to be “outside” animals or companions to be used for only specific tasks or work, such as hunting or herding farm animals. In other cultures, dogs are not kept as pets because the transmission of diseases, such as rabies, from dogs to humans is a significant concern [115].

Practitioners of some religions traditionally do not view animals as house pets. For example, people who practice the religion of Islam often do not have a favorable attitude towards dogs and having a dog as a pet is extremely uncommon in many predominately Muslim areas of the world [116]. In a study of attitudes toward pets in Kuwait, a country with a predominately Muslim population, researchers found that ratings of Kuwaiti families’ “attachment” to their pets scored approximately one standard deviation lower than the scores in a comparable study conducted in the United States [117]. Yet, there are Muslims who have countered with their own interpretation and embrace dogs [118]. Even so, employees with some religious or cultural heritages may find the prospect of sharing a workspace with a dog objectionable.

### 4.7. Welfare Concerns

Dogs may provide various benefits to employees in a workplace, but the care and welfare concerns of the animal is an important consideration [53]. Animal welfare concerns are often described using the Five Freedoms, a list of five statements describing what animals are entitled to written in 1965 in the United Kingdom by a commission addressing welfare concerns in agricultural settings [119]. The Five Freedoms are: (1) freedom from hunger or thirst, (2) freedom from discomfort, (3) freedom from pain, injury or disease, (4) freedom to express (most) normal behavior, and (5) freedom from fear and distress. These have been incorporated into the philosophies of many animal welfare organizations, including the American Society for the Prevention of Cruelty to Animals, the Association of Shelter Veterinarians, and the Federation of Veterinarians of Europe.

The welfare concerns of dogs in laboratory and shelter settings also have been formally addressed. Dogs have been used as subjects in psychological and biomedical research since at least the early 1800s [120]. Today, the use of dogs in research settings in the U.S. typically follows welfare guidelines, such as the Guide for the Care and Use of Laboratory Animals, developed by the National Research Council’s Institute for Laboratory Animal Research [121]. The guidelines specify the size, temperature, and types of the enclosures, describe appropriate types of environmental enrichment (e.g., manipulable toys), and provide recommendations for physical activity and exercise. The Association of Shelter Veterinarians (ASV) created the Guidelines for Standards of Care for Animal Shelters, which describes similar guidelines for cage sizes, enrichment, socialization, and cleaning. The ASV guidelines also describe methods to reduce zoonoses and animal-related injuries to humans.

More recently, guidance has been provided for ensuring the welfare of therapy and assistance animals [122]. Regardless of the setting, responsible pet owners, service-dog handlers, or therapy-dog handlers must be sensitive to the health and well-being of his or her dog at all times. This includes being aware of stressful environments and their effects on the dog, ensuring that the dog is allowed ample time to engage in play and other species-typical behaviors, and accommodating changes in energy levels due to old age. By making accommodation for dogs in the workplace, employers also may bear some responsibility for ensuring that animal welfare concerns are continuously addressed.

Additional welfare considerations may need to be made in work environments in which multiple dogs are present to ensure the safety of both dogs and employees. Fights between dogs have the potential to injure both the dogs and the people attempting to separate them. Dog-to-dog interactions are evaluated in the Canine Good Citizen Test [123], which is another benefit to using it to screen dogs permitted in the workplace. Workplaces may wish to implement strict on-leash guidelines and plan the work environment so that dogs are not close to one another to prevent conflict. Consulting with an applied animal behaviorist for site-specific recommendations before permitting multiple dogs in the work environment may also be advisable.

In addition to concerns about the well-being of the dog, the well-being of the service dog-handler team is an important consideration. When service dogs become a part of the work environment, employers can make a concerted effort to prepare the employees in the workplace by providing education and training on service dog etiquette and establishing and respecting boundaries for all employees. Any such interventions are typically planned with the service dog handler to respect their privacy and autonomy [53].

## 5. Considerations for Human Resource Management Personnel

Until the knowledge gaps are filled by more empirical research and reports of successes and failures, human resource personnel can carefully consider the wide range of issues that are associated with the decision to accept dogs in the workplace. The potential benefits and appropriateness of dogs in the workplace can be evaluated with respect to employee well-being, job performance, health and safety, and social interactions. Human resource management personnel may frame their policies and practices around an understanding of important differences among introducing a visitation dog, opening the workplace to employees’ pets, and accommodating an employee with a service dog. Exploratory research on service dogs in the workplace has called for caution in implementing employment site policies and procedures [124]. A service dog team can work directly with the employer to ameliorate the concerns in the process of requesting an accommodation for a dog to assist with essential functions of the job. It is important to protect privacy and minimize potential stigma associated with disclosure of details related to a person’s disability and avoid policies that would create undue hardship for an employee [53]. For the employee with a disability, the dog is not a pet or in the workplace for the benefit of all employees but a working dog that is part of an essential support system.

Based on the current, albeit limited evidence base, we offer the following specific considerations for human resource personnel tasked with addressing the inclusion of pets in the workplace in six topic areas of diversity, employee relations, ethics and corporate responsibility, organizational and employee development, safety and security, and legal policy (see Table 1). Related to issues of diversity, it is important to recognize the cultural or religious perspectives of employees toward dogs and, if possible, seek appropriate accommodations for their beliefs and preferences. This can be accomplished only if employers are informed. Thus, we suggest that employers develop a procedure to assess employees’ attitudes, beliefs, and opinions concerning the presence of dogs in the workplace. The method of assessment can be formal (e.g., surveys) or informal (e.g., focus groups), but conducting them regularly can help capture any changes in employee perceptions. Administering a “pet attitude” questionnaire, such as the Pet Attitude Scale [125] or the Measure of Human–Animal Bond [126], may be a useful tool to assess an individual’s attitudes towards dogs and other animals. Additionally, an article by Cash and Gray [127] provides a framework for accommodating different religions in the workplace that may be helpful.

Allowing pets in the workplace undoubtedly affects employee relations. To avoid potential conflicts, it is important to devise a strategy for incorporating employee input into decisions involving dogs in the workplace. Some employees may be hesitant to express their reservations or concerns because of fear of judgment by other employees, so an avenue for anonymous submission of feedback may be appropriate. One approach for handling concerns that has been adopted at some pet-friendly workplaces where there are multiple pet dogs present is the creation of a “Dog Committee”, which is a special committee of employees to evaluate issues and concerns related to the dogs as they arise. Milgate et al. [135] describe best practices for safety committees based on the results of a literature review, and their recommendations may be helpful in defining the role and duties of a workplace dog committee. Some of their recommendations include strong management commitment, effective communication among all stakeholders, and input from a health and safety professional. Additionally, to accommodate workers with dog-related fears or phobias, employers could provide them with an opportunity to privately disclose their fears or anxiety to their supervisors or human resources personnel. Accordingly, the employer may wish to designate “dog-free” areas to ease the anxiety of some individuals. The employer or human resource managers will need to determine at what point the objections of one or more employees to the inclusion of dogs are sufficient to limit or exclude dogs from the workplace.

Ethical and animal welfare concerns are important considerations for companies. Accordingly, the development of policies and procedures for the treatment and care of dogs in the workplace is an important step towards ensuring that dogs are treated humanely. This holds true for service dogs and would support successful integration of a service dog team [53]. Although “best practices” for the development of such guidelines have yet to be elucidated, the process can involve collaboration among all stakeholders in an organization, including executives, managers, supervisors, employees (especially if the employee is a service dog handler), and (if appropriate) customers. Although published guidance specific to the care of dogs in pet-friendly workplaces is limited, there are resources from other arenas that may be helpful. The Guide for the Care and Use of Laboratory Animals may be helpful in understanding humane and accepted practices and standards to ensure animal welfare. Although The Guide and other similar resources are intended for research and laboratory settings, they address many of the important animal welfare and employee health and safety concerns. Additionally, welfare guidelines for therapy and assistance animals may also aid in the development of policies and procedures [122]. To decrease the risk of dog bites or other safety and health risk caused by ill-mannered dogs, employers may wish to require that all pet dogs pass a temperament test, such as the American Kennel Club’s Canine Good Citizen Test [123]. Dogs that pass the test have demonstrated to a certified evaluator that it can calmly accept a friendly stranger, walk on a loose leash, and remain calm when separated from its owner.

The popularity of pet-friendly workplaces seems to hinge on expectations of positive benefits to employee morale and well-being. However, until these expectations are confirmed through actual experience or empirical research studies, employers may consider implementing a system for regularly assessing the impact of their own policies. We suggest that companies use anonymous surveys, focus groups, interviews, direct observations, or other methods to help assess and monitor over time the impact of dogs in the workplace. These tools or methods could assess a broad range of important employee and organizational outcomes, such as employee attitudes, perceptions, and morale, employee performance and productivity, frequency, duration, and quality of interpersonal and social interactions, absenteeism, and turnover rates. Furthermore, assessment of these outcomes can inform a continuous improvement process, which we suggest to address any concerns that arise and to promote or maximize the overall effectiveness of the program at both employee and organizational levels. The National Institute for Occupational Safety and Health (NIOSH)’s Total Worker Health website offers a list of resources for implementing and evaluating workplace health programs [131]. Resources include the U.S. Centers for Disease Control and Prevention National Healthy Worksite Program (NHWP) Health and Safety Climate Survey (INPUTS) [136], a short survey used to assess employee perceptions of the work environment, and the Center for Promotion and Health in the New England Workplace Healthy Workplace Participatory Program [137], an interactive assessment and planning process for workplace health programs, among others.

The safety and security of employees is always a high priority, thus it is important to establish policies and procedures to address employee health and safety concerns associated with dogs in the workplace. These policies and procedures could identify and describe the potential hazards and risks, specify appropriate safety control measures, and identify whether any special skills or employee training is required. At a minimum, we would suggest specific procedures to help prevent or reduce incidence of slips, trips, and falls in areas where dogs are permitted, reduce the risks of zoonotic disease transmission (e.g., require the owners to administer monthly flea and tick treatments), and facilitate the safe evacuation of employees and dogs during emergencies. The Occupational Safety and Health Administration (OSHA)’s website has guidance on OSHA standards, hazards and solutions, and hazard evaluation that may be helpful [132]. For example, employers can use the Hazard Identification Training Tool to help them learn the core concepts of hazard identification. Training materials for dog owners could be developed to ensure that they are educated on their responsibilities in maintaining a safe and healthy work environment.

Because the adoption of dog-friendly policies can pose several questions and concerns about legal liabilities, it is important to understand the federal, state, and local laws regarding the presences of dogs in public spaces in addition to the accommodation of service animals for individuals with disabilities. Important considerations include ensuring that all parties carry appropriate liability insurance policies and ensuring that dog owners have complied with all state- or municipality-mandated taxes, vaccinations, and identification for their dogs before granting them access to the workplace. Awareness of applicable leash and identification laws in the state or municipality are also critical, and employees would benefit from receiving clear guidelines for handling the dog when they are coming in and out of work and when taking their dogs outside for breaks. To ensure that practices related to considering requests for accommodation of a service dog are compliant with relevant laws, we suggest that employers seek legal counsel or contact the Job Accommodation Network, a federally-funded resource that offers free guidance to employers [133].

## 6. Knowledge Gaps and Future Research Directions

Although there may be benefits to allowing dogs in the workplace, many questions still remain about the short- and long-term effects of dogs in work settings. Efforts to bridge the knowledge gaps in these areas will help employers and human resource personnel make informed decisions about the potential benefits and challenges of accommodating dogs in the workplace. The issue is complex and requires the efforts of multiple professional and scientific disciplines. A list of knowledge gaps and suggested research topics for each discipline appears in Table 2. Because the presence of a dog in the workplace is likely to impact organizational policies, business functions, and interpersonal dynamics, human resource management personnel will be central to decision and policy making. Perhaps the greatest need is for tools that are designed to assess the impact of dogs in the workplace on employees or business operations. Currently, there are few, if any, tools or methods specifically designed to assess the impact of dogs on organizational operations and productivity, employee attitudes and perceptions, or employee social interactions. Another fruitful topic for research is to review and analyze the experiences of organizations that allow dogs in the workplace. A better accounting of these experiences across many different sizes and types of business can help employers and human resource personnel benchmark successful organizational policies, procedures, and practices.

The addition of dogs to a work setting can also create concerns related to occupational health and safety, such as potential disease transmission from animals to humans and the introduction of new workplace hazards and risks (e.g., bites, slips, trips, and falls). Because little information is available on the incidence or prevalence of zoonotic disease transmission in workplaces with pet-friendly policies, researchers should conduct targeted surveillance and epidemiological research studies on the safety and health impact of dogs in the workplace. Also needed are evaluations of existing company practices and policies regarding prophylactic measures implemented by employers to prevent zoonoses, such as regular flea and tick treatments, veterinary evaluation of stool samples for parasites, regular vaccinations, and other such protocols. Research is also needed to document the incidence and prevalence of injury-related hazards and risks associated with dogs in the workplace. Surveillance and epidemiology studies can help to reveal the nature and extent of any adverse safety and health consequences and evaluate the effectiveness of various engineering or administrative controls for reducing the hazards and risks associated with dogs in the workplace.

Seeking a better understanding of the human–animal bond and the many purported psychosocial and organizational benefits of dogs or other pets in the workplace offers other fruitful areas for research. There have been a large number of studies conducted on the effects of dogs on the mental health of children [138], older adults [89], and patients in psychiatric care settings [19], but there has been comparatively less research conducted on the effects of dogs on adult well-being in the workplace. More research studies are needed to understand the impact of dog-friendly policies on employees both before and after the introduction of dogs on measures of perceived stress and work load, performance and productivity, employee relations, organizational climate/culture, absenteeism, and other employee and organizational outcomes. Another research question concerns the long-term maintenance of any positive effects of dogs in the workplace. Some have raised the notion that the introduction of dogs produces only short-term improvements in health and well-being, and that the positive aspects dissipate as the novelty of the dogs’ presence wears off [139]. Longitudinal studies, in which impact is assessed over time, would help to address this question. A final research question concerns the impact of dog-friendly corporate policies on public perceptions. The reporting of pet-friendly corporate policies of companies in the popular press [140] has perhaps contributed to positive public reactions, but more research is needed to compare similar businesses with and without dog-friendly policies to better understand whether these policies support a competitive advantage, not only in relation to consumer preferences but also by attracting and retaining the most qualified employees.

## 7. Conclusions

Given that many individuals in today’s workforce struggle with long work hours, increasing job demands, and high stress, the notion of dog-friendly work settings to help mitigate these stressors has great appeal to both employees and employers. Indeed, the benefits of dog-friendly work places may manifest as lower rates of absenteeism and higher worker morale and productivity. Beyond employers’ broad desires to create a pet-friendly work environment, employers who are only accommodating service dogs would be well-served to do so with care and consideration for all parties involved. Although several knowledge gaps exist concerning specific benefits, concerns, and challenges associated with accommodating dogs in the workplace, employers and human resource personnel can attempt to weigh the potential benefits of welcoming dogs in the workplace with the related health, safety, psychosocial, and animal welfare concerns using available resources. The decision to accept dogs or other animals into a work setting is complex and ought to be made with careful consideration of all relevant factors.

## Figures and Tables

**Table 1:** Considerations and suggested resources for additional guidance for accommodating dogs in the workplace.

Topic Area	Considerations	Resources
Diversity	Establish formal or informal procedures for periodically assessing employee attitudes, beliefs and preferences concerning dogs in the workplace. Develop policies and procedures for accommodating employees with special concerns (e.g., cultural/religious beliefs, fears, or phobias) in a sensitive and confidential manner.	Cash and Gray [127]
Employee Relations	Form a “Dog Committee” with employees and management to provide a forum in which employee questions and concerns related to dogs in the work environment can be addressed.	Morse, et al. [128]
Ethics and Corporate Responsibility	Establish temperament/behavioral requirements for dogs (e.g., passing the AKC Canine Good Citizen test or similar assessment). Develop policies and procedures for the treatment and care of dogs while in the workplace; explicate the responsibilities of the dog owner and the employer.	American Kennel Club [123] National Research Council [129] Newbury, et al. [130] Serpell, et al. [122]
Organizational and Employee Development	Establish formal or informal procedures for evaluating the impact of dogs in the workplace on employee morale, well-being, productivity, etc. Establish a continuous improvement process to address concerns and maximize the effectiveness of the dog program.	The National Institute for Occupational Safety and Health [131]
Safety and Security	Develop policies and procedures to help prevent or reduce the incidence of slips, trips and falls in dog areas. Develop training materials to ensure that dog owners are educated on their responsibilities in maintaining a safe and healthy work environment.	Occupational Safety and Health Administration [132]
Legal Considerations	Understand the federal, state, and local laws regarding accommodating individuals with disabilities and their service dogs. Understand the state and local laws regarding identification, vaccination, and leashing for pet dogs. Establish clear eligibility criteria for both dogs and their employee owners. For example, specify specific training requirements for employees and breed/heath/behavioral requirements for dogs. Seek legal counsel to review all policies and procedures concerning the accommodation of dogs in the workplace.	Job Accommodation Network [133] Michigan State University School of Law [134]

**Table 2:** Knowledge gaps and suggested research topics related to the accommodation of dogs in the workplace.

Focus Area	Research Topics
Human Resource Management	Develop tools (e.g., surveys or interviews) for assessing the potential impact of dogs in the workplace on employees and operations. Develop tools (e.g., surveys or interviews) for assessing the attitudes and perceptions of employees in dog-friendly workplaces. Assess what policies and practices are being implemented in dog-friendly workplaces to accommodate employees with dog fears or phobias. Assess what policies and practices are being implemented in workplaces that permit dogs but also have employees with pet dander allergies. Assess the impact of the presence of dogs in the workplace on employee relations.
Occupational Health and Safety	Conduct surveillance or epidemiological research studies to document the prevalence and severity of injuries and zoonotic diseases in workplace settings where pets are present. Evaluate the effectiveness of safety and health precautions currently being implemented by workplaces that permit animals and identify and best practices. Develop tools or methods that can be used to assess the hazards, risks, and impact of dogs in the workplace. Identify/assess the potential hazards and risks of dogs in the workplace, including the risk of zoonotic diseases. Identify and evaluate specific engineering or administrative controls that reduce the hazards and risks associated with dogs in the workplace. Evaluate the effectiveness of specific safety and health controls (e.g., high-efficiency particulate air (HEPA) filters to reduce dog dander in workplace settings).
Behavioral and Social Science	Investigate the impact of the presence of dogs in the workplace on employee morale, productivity, and well-being. Examine whether dogs in the workplace improve the attitudes, mood, perceptions, and performance of employees. Investigate the effect of dog-friendly work policies on organization climate/culture and employees‘ perceptions of the organization and working conditions. Evaluate the effects of dog-friendly policies on the public‘s perceptions of the company.
